# The application of local hypobaric pressure — A novel means to enhance macromolecule entry into the skin

**DOI:** 10.1016/j.jconrel.2016.01.052

**Published:** 2016-03-28

**Authors:** R. Inacio, S. Poland, X.J. Cai, S.J. Cleary, S. Ameer-Beg, J. Keeble, S.A. Jones

**Affiliations:** a King's College London, Faculty of Life Sciences & Medicine, Institute of Pharmaceutical Science, Franklin-Wilkins Building, 150 Stamford Street, London SE1 9NH, United Kingdom; b King's College London, Division of Cancer Studies and Randall Division of Cell & Molecular Biophysics, Guy's Medical School Campus, London SE1 1UL, United Kingdom

**Keywords:** Hypobaric pressure, Macromolecule, Skin, Follicle, *In vivo*, Blood flow, Bioavailability, Drug delivery, Penetration enhancement

## Abstract

The local application of controlled hypobaric stress represents a novel means to facilitate drug delivery into the skin. The aims of this work were to understand how hypobaric stress modified the properties of the skin and assess if this penetration enhancement strategy could improve the percutaneous penetration of a macromolecule. Measurements of skin thickness demonstrated that the topical application of hypobaric stress thinned the tissue (*p* < 0.05), atomic force microscopy showed that it shrunk the corneocytes in the *stratum corneum* (*p* < 0.001) and the imaging of the skin hair follicles using multiphoton microscopy showed that it opened the follicular infundibula (*p* < 0.001). Together, these changes contributed to a 19.6-fold increase in *in vitro* percutaneous penetration of a 10,000 molecular weight dextran molecule, which was shown using fluorescence microscopy to be localized around the hair follicles, when applied to the skin using hypobaric stress. *In vivo*, in the rat, a local hemodynamic response (*i.e.* a significant increase in blood flow, *p* < 0.001) was shown to contribute to the increase in follicular transport of the dextran to produce a systemic absorption of 7.2 ± 2.81 fg·mL^− 1^. When hypobaric stress was not applied to the rat there was no detectable absorption of dextran and this provided evidence that this novel penetration enhancement technique can improve the percutaneous penetration of macromolecules after topical application to the skin.

## Graphical abstract

**Figure:**
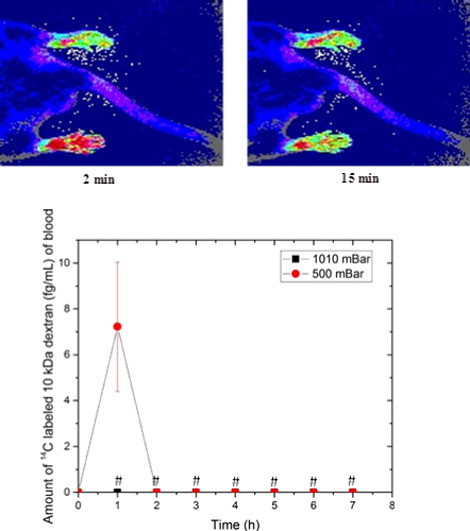

## 1 Introduction

The delivery of macromolecules such as peptides, proteins and antibodies *via* the skin is an attractive proposal when considering the treatment and prevention of diseases. However, their administration *via* this route is a challenge due to the skin's highly stratified structure [1], [2]. The *stratum corneum* and the multiple underlying layers, which constitute the cutaneous tissue, exhibit highly selective permeability that only allows the rapid absorption of relatively small, lipophilic compounds to enter the body after topical administration (< 500 Da, Log P 0.8–3) [3], [4]. Therefore, the only means by which macromolecules can enter the skin tissue at a rate that is suitable for therapeutic applications is by the use of a penetration enhancement strategy [5], [6], [7], [8], [9]. While many of the developed approaches to allow macromolecules into the skin are effective they each have associated cost, compliance and safety issues that make their use limited in clinical practice [10]. There therefore remains a need to develop new technologies to facilitate the passage of high molecular weight therapeutic agents into the skin.

The few documented reports that have assessed the capability of applying local hypobaric stress to alter the properties of the skin suggest that it could represent a novel means to enhance drug percutaneous penetration. For example, the application of local hypobaric stress has been documented to significantly increased transepidermal water loss (TEWL) and decrease the *stratum corneum*'s water content [11]. These effects could potentially thin the skin and facilitate drug entry. In addition, the hypobaric stress induced stretching of the skin has been shown to cause disorganization of the intercellular lipid bilayers and rupture of the skin's corneosomes [12], [13]. Increasing the lipid fluidity is a mechanism that chemical penetration enhancers use to increase percutaneous penetration and therefore this could be an important means by which hypobaric stress could act to enhance percutaneous penetration. Furthermore, the continuous application of localized sub-atmospheric pressures has been reported to significantly increase cutaneous blood flow [14], [15]. Increasing the blood flow under local hypobaric stress conditions may increase the systemic absorption of topically applied agents due to the dilation of the dermal blood vessels and their displacement towards the skin surface [16]. However, how these effects of hypobaric stress influence skin penetration of macromolecules is currently unknown.

The purpose of the present study was to assess the influence of locally applied hypobaric stress upon the mechanical and physiological properties of cutaneous tissue and then using this knowledge test if hypobaric stress could enhance the percutaneous penetration of macromolecules. To apply controlled hypobaric stress to the skin one of the most widely used test systems for studying skin permeability, the Franz diffusion cell, was adapted to operate at a sub-atmospheric pressure of 500 mBar. A series of fluorescein isothiocyanate (FITC)-labeled dextrans was used as the model macromolecules for this work because of their molecular homogeneity, their excellent chemical and physical stability, their commercial availability and their ability to be tracked *in vitro* and *in vivo* [17]. *In vitro* permeation studies were performed using rat skin. It is accepted that rat skin is more permeable compared to human skin, but this membrane was selected primarily because this allowed similar studies to be performed *in vitro and in vivo*. In addition, the rat was easily amenable to the measurement of blood flow using full-field laser perfusion imaging (FLPI) and systemic bioavailability using standard blood sampling protocols [18], [19], [20]. Porcine skin was also utilized as a permeation barrier to explore the transdermal transport mechanism of the macromolecules because it is the most relevant animal model for human skin [18]. In addition, this barrier is especially suitable for studying follicular structures as the ear cartilage prevents contraction of tensile fibres and closure of follicles upon sample preparation, which can occur with human and rat skin [21]. The changes in the skin caused by the application of hypobaric stress were assessed using multiphoton microscopy, light microscopy and atomic force microscopic (AFM) imaging. In addition, the entry of the dextran into the cutaneous tissue was followed using fluorescence microscopy to try and elucidate the route of entry of this molecule into the skin.

## 2 Materials and methods

### 2.1 Materials

Acetonitrile and methanol both HPLC grade, DPX mounting medium, xylene and Optiphase scintisafe gel were purchased from Fischer Scientific (Leicester, UK). Tetracaine base BP grade (99.9%), formalin solution neutral buffer 10%, DAPI medium, ethanol, heparin sodium salt (I-A), urethane, 0.7% glacial acetic acid, isopropanol and FTIC-dextran with average molecular weight (Mw) of 4 kDa (FD-4) and 10 kDa (FD-10S) used without any further purification steps were supplied by Sigma Aldrich (Dorset, UK). Concentrated hydrochloric acid and sodium hydroxide was from Fluka (Dorset, UK). Sodium acetate was provided by Alfa Aesar (Heysham, UK). Silicone membranes with a thickness of 0.25 mm were purchased from GBUK Healthcare (Selby, UK). Phosphate buffered saline (Dulbecco A) tablets were obtained from Oxiod Limited (Hampshire, England). The Tissue-Tek® O.C.T™ compound, scintillation vials and hydrogen peroxide 30% were obtained from VWR International (Leicestershire, UK). Dextran (carboxyl-^14^C) with an average M.W. of 10 kDa and specific activity of 0.00006 Ci/mmol was obtained from American Radiolabeled Chemicals, Inc. (St. Louis, USA). Soluene® 350 was provided by Perkin Elmer (Bucks, UK). Isoflurane 100% (w/v) inhalation vapour liquid was obtained from Animal Care Ltd. (York, UK).

### 2.2 Animals

All procedures were conducted in accordance with the UK Animal Scientific Procedures Act (1986) and Amendments Regulations (2012) and approved by the King’s College London Animal Welfare and Ethical Review Body. Sprague Dawley male rats (6–9 weeks old, *ca*. 220–250 g; Charles River, Kent, UK) were caged in groups of 4 with free access to water and food. A temperature of 19–22 °C was maintained, with a relative humidity of 45–65%, and a 12 h light/dark cycle. Animals were acclimatized for 7 days before each experiment.

### 2.3 *In vitro* dextran permeation studies

Rats were killed by intraperitoneal injection of sodium pentobarbital. The dorsal hair was removed using an animal hair clipper and full thickness skin was excised. The excess fat adhering to the dermis side was removed carefully with a scalpel. A standard Franz diffusion cell was attached to an in-house designed aluminium support frame (Supplementary data Fig. S1a, b and c) that was able to pressure seal the donor compartment (Supplementary data Fig. S1d). To develop a sound experimental protocol using the assembled pressure cell set up, a series of transport experiments with porcine skin were conducted. These preliminary studies indicated that the receiver chamber of the Franz cell could not be filled with a liquid because the hypobaric pressure caused suction of the liquid from the donor phase into the receiver compartment. As a consequence, the traditional Franz cell was adapted to use a sponge in order to collect the drug exiting the dermal side of the skin. The sponge was shown to remain in contact with the skin through the experiments and it allowed the complete recovery of the drug that had passed through the skin (Supplementary data Fig. S2). For the *in vitro* dextran permeation studies (note: fluorescein isothiocyanate (FITC)-dextran was used throughout the *in vitro* section of work), rat skin was employed using the permeation methodology developed with the porcine skin. The depilated rat skin harvested from the animals was cut into pieces of a suitable size and mounted with the *stratum corneum* facing the donor compartment in the Franz diffusion cell (University of Southampton, UK). The pressure cell sealed donor compartment was attached to the Franz cell base and each diffusion cell was placed on a submersible stirring plate in a pre-heated water bath (Grant Instruments, Cambridge, UK) set at 37 °C, to obtain a temperature of 32 °C at the membrane surface [22]. The sealing of the cells was evaluated by their resistance to air removal and the absence of significant solvent back diffusion into the donor compartment prior to the application of hypobaric pressure. Any leaking cells were not used in the experiments and hence all the data was used in the data analysis. The experiments were initiated by the application of 1 mL of a donor solution (containing either 125 μM of FD-4 or FD-10S in phosphate buffer solution at pH 7.4) to the apical surface of the skin. Permeation experiments were conducted under atmospheric (1010 mBar) and hypobaric pressure (500 mBar) for 1 h using the pressure cell assembly. The hypobaric conditions had previously been tested in a pilot study and it appeared to cause no skin blistering (data not shown), but this was investigated further using skin histology (detailed in a subsequent section). The hypobaric pressure was applied to the skin immediately after the application of the donor solution by the removal of air from the Franz cell donor compartment, which was sealed onto the skin and thus acted as a vacuum chamber. A manometer was mounted on the donor compartment in order to record the pressure applied. Hypobaric stress was applied for 1 h and any small loss in pressure was corrected by extracting further air from the Franz cell. After the 1 h time period of hypobaric stress the cells were allowed to equilibrate at atmospheric pressure and the permeation experiments were continued for a total of 20 h to allow suitable drug levels to accumulate in the skin to facilitate accurate analytical quantification. At the end of the transport studies the *stratum corneum* was removed by tape stripping (*ca.* 20 strips until the skin was translucent) using adhesive tape (Scotch 845 book tape, 3 M, Bracknell, UK) and the epidermis was separated from the dermis as previously described [23]. Dextran was extracted from the receptor compartment, adhesive tape and skin using a phosphate buffer saline (pH 7.4) extraction solution. The dextran was quantified using a stand-alone fluorescence spectrometer (Varian Cary Eclipse fluorescence spectrophotometer, Agilent, Cheadle, UK) at an excitation wavelength of 495 nm and fluorescent emission wavelength of 515 nm. The assay was verified as ‘fit for purpose’ by determination of linearity, precision and sensitivity (data not shown). Drug extraction was within the 100 ± 15% recovery rates published in guidelines [24]. The effect of local hypobaric stress upon dextran cutaneous bioavailability was represented as an enhancement ratio (*ER*), which was calculated according to Eq. (1) where *C*_*P*_ and *C*_*AT*_ were the amount of dextran (μg) per cm^2^ of skin under hypobaric and atmospheric pressure conditions, respectively.

(1) $$\mathrm{ER}=\frac{C_{P}}{C_{\mathrm{AT}}}.$$

### 2.4 Fluorescence microscopy

The skin samples remaining after the completion of the (FITC)-dextran transport studies were subject to fluorescence microscopy analysis. To perform the analysis the skin samples were prepared by cutting them in half along their diameter and embedding them in O.C.T. compound. The embedded samples were sectioned in 20 μm slices using a cryostat microtome (Bright Instruments, Huntingdon, UK). The skin sections were mounted, stained with DAPI and covered with glass cover slips. Fluorescence photomicrographs were obtained with a Zeiss Axioscope microscope equipped with a Nikon Digital Camera (DXM1200; Nikon, Kingston upon Thames, UK) at a magnification of 10 ×. Images were acquired using two fluorescence channels to allow the visualization of the cellular structures stained by DAPI (blue colour emission ~ 460 nm) and the FITC-dextran fluorescence signal (green colour emission ~ 520 nm). Tissue samples without FITC-dextran were also tested as controls. Images were processed using Image J Software (National Institutes of Health, Maryland, USA).

### 2.5 Skin morphology and physiology

#### 2.5.1 Multiphoton fluorescence microscopy analysis

Multiphoton microscope images were taken using a custom built system developed around a FN1 upright microscope (Nikon Instruments, Melville, USA). The excitation source was a femtosecond pulsed Titanium:sapphire Chameleon Vision S laser (Coherent Inc., Santa Clara, USA) tuned to 800 nm, which was relayed into an afocal galvanometer scanning system. This was then projected onto the back aperture of an infinity corrected Nikon air-objective (NA. 0.5 × 20; Nikon Instruments, Melville, USA), where it was focused on the sample. The emitted fluorescence was collected onto two detection channels housed in a non-descanned configuration, with bandpass filters of 525 ± 30 nm and 593 ± 40 nm, respectively. The detectors used were a HPM-100-50 Hybrid Photomultiplier Tubes (PMTs) (Becker & Hickl, Berlin, Germany) operating in single photon counting mode. The microscope was controlled by a software developed using the graphical based LabVIEW programming language (National Instruments Corporation, Austin, USA). For these experiments porcine skin was challenged under hypobaric pressure conditions employed in the permeation studies in order to try and retain the follicular structure (Supplementary data Fig. S2). Porcine skin was used to develop the hypobaric model and it was shown to behave in a very similar manner to rat skin upon the application of hypobaric pressure using a number of different tests in this work (see Fig. S3 for an example). After hypobaric treatment, the stressed skin was immediately mounted on a glass slide with the *stratum corneum* facing the objective. Z stacks of the porcine skin samples were acquired using Bio-Rad software (Philadelphia, USA) and were taken from the *stratum corneum* to the dermis (1–100 μm deep), with 2 μm steps. Two dimensional images were generated by raster scanning the excitation beam across the skin sample utilizing the afocal scanning system. Measurements of the hair follicle infundibula were performed using in-house developed software allowing the assessment of the length and depth of the follicular structures.

#### 2.5.2 Atomic force microscopy analysis

Corneocytes were collected from the porcine skin surface (*n* = 6), treated under the same hypobaric stress conditions employed in the permeation studies, by the removal of 4, 6 and 10 tape strips from the same cutaneous site. Porcine skin was used in the AFM studies because it allowed the collection of single corneocytes, which was required for the imaging. This method had been previously described by Fredonnet et al. [25]. The tape with the collected corneocytes was then fixed on a glass slide and images were obtained from a Nanoscope V multimode scanning atomic force microscope (Digital Instruments, Coventry, Bresso, ItalyUK). Imaging was performed in tapping mode in air. Aluminium coated Si3N4 cantilevers with integrated pyramidal tips (NSC15/Al, MikroMasch, Wetzlar, Germany) with a reported resonance frequency of 325 kHz and a spring constant of 40 N/m were used. After acquisition, the images were flattened and analysed using section analysis with the Gwyddion software (Czech Metrology Institute, Brno, Czech Republic). The cell outlines were clearly visible from the computer display and this allowed the cells size to be determined using the AFM software.

#### 2.5.3 Histological studies

Skin samples, challenged under the same hypobaric stress conditions employed in the permeation studies, were cut into small pieces and fixed with 10% neutral-buffered formalin for 24 h at room temperature. Both porcine and rat skin samples were used in these studies. These samples were subsequently embedded in O.C.T. [26]. Cross-section slices of 20 μm thickness were obtained using a Bright Model OTF cryostat (Bright Instruments, Huntingdon, UK). The samples were stained following the Ellis Haematoxylin and Eosin (H&E) staining protocol [27] and dehydrated with different volumes of ethanol (75%, 95%, and 100%) and xylene for 5 min each, before being mounted in DPX and covered with glass cover slips. The samples were analysed using a Leica DM 200 Led light microscope (Leica Microsystems, Wetzlar, Germany) equipped with a Leica digital camera (Model DFC 295) at a magnification of 4 × and 40 ×. Images were processed using Las v4.4 Imaging Software (Leica Microsystems, Wetzlar, Germany).

### 2.6 Cutaneous blood flow measurements

Rats were anaesthetized by inhalation of (~ 2%) isoflurane/(~ 2%) O_2_ and cutaneous blood flow was assessed in the whole plantar hind paw area using the Full-Field Laser Perfusion Imager (FLPI, Moor Instruments, Axminster, UK). Baseline blood flow in both hind paws was recorded prior to the application of hypobaric stress to ensure the hemodynamic vascular responses were stabilized following the induction of anaesthesia. The ipsilateral hind paw was subsequently exposed to 500 mBar of hypobaric stress for 7 min (n = 6) using the same adapted pressure cell described for the *in vitro* permeation studies, but without the receiver compartment as the cell was placed directly onto the rat's hind paw. The pressure cell application time was reduced to 7 min in the *in vivo* studies because this was the minimum time that was shown in pilot studies to change blood flow. The contralateral paw was untreated and served as a control. Changes in blood flow after hypobaric stress treatment were followed for 15 min. The rats were placed on a heating mat (Harvard Apparatus, Cambridge, UK) in the ventral position maintained at 36 °C for blood flow measurements. All animals were culled upon termination of the experiment. Results were expressed as a measure of % change in blood flow from baseline. Eq. (2) was used to drive % change in blood flow and Eq. (3) was used to calculate the maximum vasodilatation.

(2) $$\%\text{Change}\;\mathrm{in}\;\text{blood}\;\text{flow}=\frac{\left(\mathrm{Blood}\;\mathrm{flow}-\text{Baseline}\right)}{\mathrm{Baseline}}\times 100$$

(3) $$\mathrm{Maximum}\;\mathrm{vasodilatation}=\frac{\left(\mathrm{Peak}\;\mathrm{vasodilatation}-\text{Baseline}\right)}{\mathrm{Baseline}}\times 100.$$

### 2.7 Pharmacokinetic studies

Animals were anaesthetized by intraperitoneal injection of urethane (0.175 g/100 g) and placed on a heating mat (Harvard Apparatus, Cambridge, UK) in the ventral position maintained at 36 °C for the duration of the experiments. The dorsal fur was carefully removed with an animal hair clipper and the donor compartment of a Franz cell with an available area of 2.1 ± 0.2 cm^2^ was attached to the shaved skin with glue [28]. Rats were bled by tail veil puncture and *ca*. 100 μL of blood was collected in a heparinized tube. To initiate the permeation studies a 300 μL aliquot of ^14^C-labeled dextran in phosphate buffer (0.79 μCi equivalent to 1.428 pM) was added to the donor compartment of the Franz cell. Permeation studies were conducted under atmospheric pressure (1010 mBar) and hypobaric pressure (500 mBar) for 7 h (*n* = 5). To apply the hypobaric pressure the pressure cell assembly was attached to the top of the Franz cell donor compartment and a sub-atmospheric pressure of 500 mBar was applied for the first hour (*n* = 5) of the experimental period. At hourly time points for 7 h, the rats were bled by tail vein puncture and *ca*. 100 μL of blood was collected to a heparinized tube. Blood withdrawn did not exceed 10% of the 6.86 ± 0.53 mL/100 g rat blood volume per day [29]. All animals were culled upon termination of the experiment. Blood samples were transferred to 20 mL scintillation vials, 1 mL of tissue solubilizer was added and the samples were shaken overnight at 55 °C. Before adding the scintillation cocktail, samples were decolorized by adding 0.3 mL of 30% H_2_O_2_ and isopropanol as an antifoaming agent. Samples were subsequently shaken at 55 °C for at least 3 h to expel H_2_O_2_ content and then mixed with 20 mL of undiluted scintillation cocktail acidified with glacial acetic acid to eliminate any chemiluminescence. The samples were stored in the dark for 24 h before counting [30]. ^14^C radioactivity was quantified for each sample using a LS6500 multi-purpose scintillation counter (Beckman Coulter, Brea, USA) with a defined limit of detection of 3 × background level measurements. Total radioactivity in the blood was calculated based on the total blood volume of 6.86 ± 0.53 ml/100 g previously reported for Sprague Dawley male rats [29]. The results were expressed as amount of ^14^C-dextran (fg) per mL of blood.

### 2.8 Cutaneous bioavailability and tissue distribution

At the end of the transport studies (Section 2.3), the dorsal skin underneath the Franz cell donor compartment and organs (heart, bladder, kidneys, liver and spleen) were collected, rinsed with distilled water and weighed. The *stratum corneum* was removed by tape stripping [23] and the adhesive strips were dissolved in 15 mL of tissue solubilizer and left at room temperature for 2 days [31]. A 2 mL aliquot of the tissue extraction solution was then transferred to 20 mL scintillation vials and mixed with 15 mL of scintillation cocktail, acidified with glacial acetic acid, to eliminate any chemiluminescence [30]. The stripped skin and whole organs were homogenized using a tissue homogenizer (Ultra Turrax, Fisher Scientific, Leicester, UK) in phosphate buffer (0.2 mL per 100 mg of tissue). A 200 μL aliquot of the homogenized solution was transferred using a positive displacement pipette to 20 mL scintillation vials, 1 mL of tissue solubilizer was added and the samples were shaken overnight at 55 °C. The radioactivity of the samples was determined as described above. Drug extraction was within the 100 ± 15% recovery rates, which was in line with published regulatory guidelines [24]. The results were expressed as amount of ^14^C-dextran (fM) per cm^2^ or per g of tissue (*n* = 5). The effect of local hypobaric stress upon ^14^C-labeled dextran cutaneous bioavailability and organ uptake was represented by an enhancement ratio (ER) which was calculated according to Eq. (1).

### 2.9 Statistical analysis

Statistical evaluation was carried out using the Statistical Package for the Social Sciences software (SPSs version 16.0, SPSS Inc., Chicago, USA). Data were checked in terms of normality (Kolmogorov–Smirnov test) and homogeneity of variances (Levene's test) prior to analysis. Statistical comparison was performed using Student's *t*-test and a statistically significant difference was defined as when *p* < 0.05 and denoted as: *p < 0.05, **p < 0.01 and ***p < 0.001.

## 3 Results and discussion

### 3.1 *In vitro* effects of local hypobaric stress

**Fig. 1:**
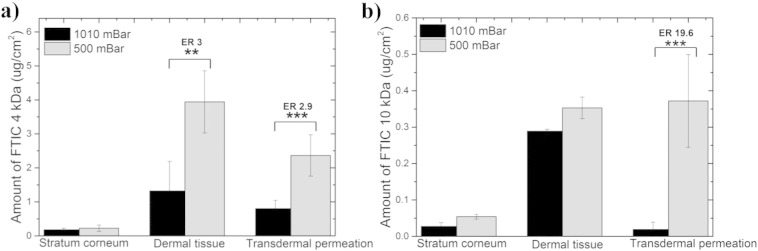
Permeation of FD-4 (a) and FD-10S (b) into rat skin under atmospheric (1010 mBar) and hypobaric conditions (500 mBar). Each point represents mean ± standard deviation (n = 4) for atmospheric conditions and (n = 5) for hypobaric conditions. ER (Enhancement Ratio) represents the ratio between the amount of drug in the tissue under hypobaric and atmospheric conditions. Student's *t*-test with **p < 0.01 and ***p < 0.001.

The application of topical hypobaric stress altered the skin deposition of both FD-4 and FD-10S (Fig. 1a). However, the different sized dextran molecules showed a different skin deposition profile in response to the application of hypobaric stress. For example, the FD-4 dextran demonstrated a 3-fold greater deposition within the dermal tissue whereas, the amount of FD-10S retained in the dermal tissue was found to be equivalent (*p* < 0.05) under both permeation conditions (Fig. 1b). The transdermal delivery of both FDs was found to be significantly greater (*p* < 0.01) when compared to atmospheric conditions (2.9 and 19.6-fold increase, respectively) (Fig. 1). However, calculating the enhancement ratios showed that the effect of hypobaric stress was more pronounced upon the transdermal delivery of the higher molecular weight dextran (10 kDa).

The change in dextran skin deposition profiles upon the application of hypobaric stress recorded in this study was not thought to be influenced by chemical degradation since it has been previously shown that FDs, of various molecular weights, were chemically stable after the permeation across rat skin under similar experimental conditions [32]. Therefore, the change in how dextran deposited in the skin was thought to be consequence of the manner in which the hypobaric stress altered the properties of the tissue. Molecules with a molecular weight of > 1000 find it problematic to pass through the skin passively *via* the inter or intracellular pathways [33]. Previously, permeation studies using FDs through rat skin, porcine skin and *in vitro* cultured human epidermis models have suggested that the follicular route is the primary permeation pathway for dextrans with a size between 4 and 10 kDa [33], [34], [35]. This was recently confirmed in a novel follicle-plugging method, which showed that the permeation of FD-4 was significantly influenced by the availability of “unplugged” hair follicles [36]. Therefore, the permeation data was thought to show the manner in which the hypobaric stress had modified the transfollicular route of transport into the skin. The superior enhancement of the FD-10S compared to the FD-4 by hypobaric stress supported this hypothesis as the former was more likely to be transported mainly through the follicular route. However, it was thought necessary to investigate this result further by trying to determine how these molecules entered the skin using fluorescence microscopy.

**Fig. 2:**
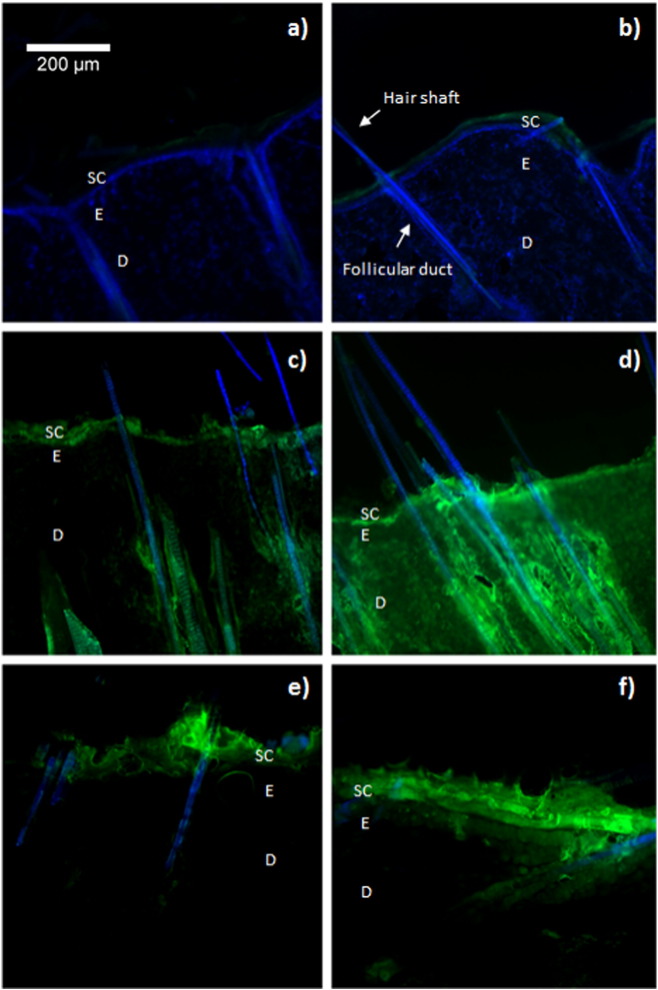
Fluorescence microscopic examination after topical administration of FD-4 and FD-10S dextran under atmospheric conditions (1010 mBar) and hypobaric stress conditions (500 mBar): control samples at atmospheric (a) and after hypobaric treatment (b); topical FD-4 delivery under atmospheric conditions (c) and upon hypobaric stress treatment (d); topical FD-10S delivery under atmospheric conditions (e) and upon hypobaric stress treatment (f). Green: FITC, blue: DAPI. Original magnification × 10. SC, stratum corneum; E, epidermis and D, dermis.

**Fig. 3:**
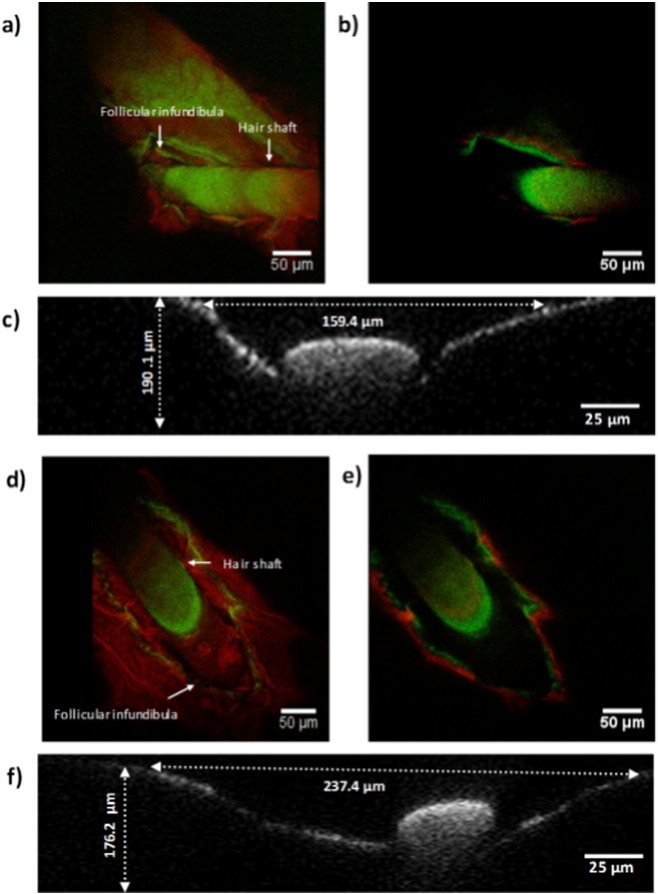
Multiphoton microscopic images of porcine follicular infundibula. a) 3D reconstruction under atmospheric conditions (1010 mBar), b) top view (singe Z-stack cross-section at 1010 mBar), c) side view at 1010 mBar with an average length of 151 ± 40.5 μm and depth of 190 ± 30.1 μm, d) 3D reconstruction after applying hypobaric pressure (500 mBar), e) top view after hypobaric pressure (Z-stack cross-section), f) side view after hypobaric pressure with an average length of 243 ± 23.9 μm and depth of 159 ± 14.5 μm.

The FITC-derived fluorescence signal appeared to be much more intense in deeper skin layers exposed to hypobaric treatment (Fig. 2d and f) compared to the samples under atmospheric conditions (Fig. 2c and e). The fluorescence signals were most concentrated around the perifollicular region for both (FITC)-dextrans, which suggested that hypobaric treatment influenced drug transport across the hair follicle structures. However, the two different molecular weight dextrans did show different skin deposition profiles upon the application of hypobaric pressure. The lower molecular weight FD dextran gave a broad and continuous spatial distribution of the fluorescence signal that extended from the *stratum corneum* into deeper layers of the skin (Fig. 2d). This implied that the FD-4 dextran was transported into the skin not only through the follicular route, but also to some extent by inter and intra cellular passive diffusion. The FD-10S only appeared in the deeper tissue layers around the perifollicular region, suggesting that the follicular pathway was the main route utilized by this molecule to penetrate into the hypobaric stressed skin. The green filter autofluorescence observed from the control samples, where no FITC-dextran had been applied (Fig. 2a and b), was minimal compared to signal from samples treated with FITC-dextran [37]. Therefore, it was concluded from theflorescence microscopy studies that the FITC-dextrans were being primarily transported by the follicular route. This supported the transport data despite there being evidence of some FD-4 passive transport through the stratum corneum. However, this data did not demonstrate how hypobaric pressure modified the hair follicle structure and so multiphoton microscopy was employed to try and investigate this aspect in subsequent experiments. The multiphoton microscopy images of the hair follicles showed that follicular infundibula of the hypobaric stressed skin were significantly wider (*p* < 0.001) than the untreated skin. An average horizontal planner length of 243 ± 23.9 μm was recorded after hypobaric pressure treatment compared to 151 ± 40.5 μm after identical sample treatment under atmospheric conditions (Fig. 3c and f). There was also a significant decrease (*p* < 0.01) in the depth of the follicular structures after the application of hypobaric stress (159 ± 14.5 μm — hypobaric pressure *vs*. 190 ± 30.1 μm — atmospheric pressure) (Fig. 3c and f). This greater horizontal planner length of the follicular infundibula with the concomitant decrease in the depth of the follicular structure was believed to be due to the stretching of the skin when subjected to hypobaric stress (Supplementary data Fig. S3b). Previous studies have suggested that the application of hypobaric pressure can be used to ‘open’ hair follicles [38] and this opening up of the follicles, which have been shown to be only partially accessible to drug permeation in their native state [39], [40], [41], was considered to be a major factor in facilitating the macromolecule percutaneous penetration when hypobaric pressure was applied to the skin in the current work. This did not however explain the increased passive inter and intracellular diffusion observed with the 4 kDa dextran and hence AFM was used to investigate the corneocytes of the *stratum corneum*.

**Fig. 4:**
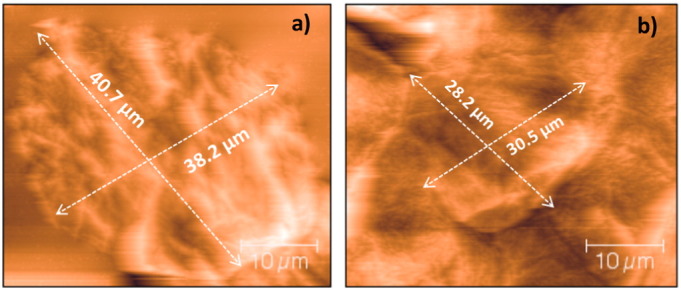
Atomic force microscopy analysis of *in vitro* porcine skin corneocytes at a) atmospheric conditions (1010 mBar) with average length of 41.5 ± 5.5 μm and width of 37.2 ± 7.2 μm and b) within 25 min of applying hypobaric pressure (500 mBar) with average length of 31.5 ± 8.2 μm and width of 26.2 ± 6.8 μm.

The AFM studies demonstrated that the average size of the porcine corneocytes was significantly smaller upon hypobaric treatment (Fig. 4). They displayed a length and width of 31.5 ± 8.2 μm and 26.2 ± 6.8 μm under hypobaric conditions *vs*. 41.5 ± 5.5 μm and 37.2 ± 7.2 μm under atmospheric conditions respectively (*p* < 0.001, n = 20). The barrier characteristics of the skin are influenced by the properties of the lipids and the path-length for diffusion. The latter depends on the number of corneocytes, their cohesion and their size. Previously, a reduction in the corneocytes packing has been shown to give rise to a concomitant increase in the size of corneocytes gaps and this was thought to enhance percutaneous penetration [42], [43]. Hence, the shrinkage of the corneocytes due to the application of hypobaric pressure was considered to be the reason for the 4 kDa dextran entering the skin more easily *via* non-follicular routes [44], [45].

The hypobaric stressed porcine skin was found to be significantly thinner at the end of the transport studies. A skin thickness of 0.89 ± 0.1 mm upon the application of hypobaric treatment *vs*. 1.1 ± 0.1 mm under atmospheric pressure conditions was registered (*p* < 0.05, n = 5). Conversely, when rat skin was treated with the same hypobaric conditions used in the *in vitro* permeation studies and subsequent *in vivo* experiments there was no significant alteration (*p* > 0.05) in membrane thickness (Supplementary data Fig. S3). Skin thinning upon the application of hypobaric stress has been previously reported in the literature [46], but as the rat skin did not show a significant thinning effect in this study it was not thought to be a major contributor to the enhanced percutaneous penetration induced by the application of hypobaric pressure.

**Fig. 5:**
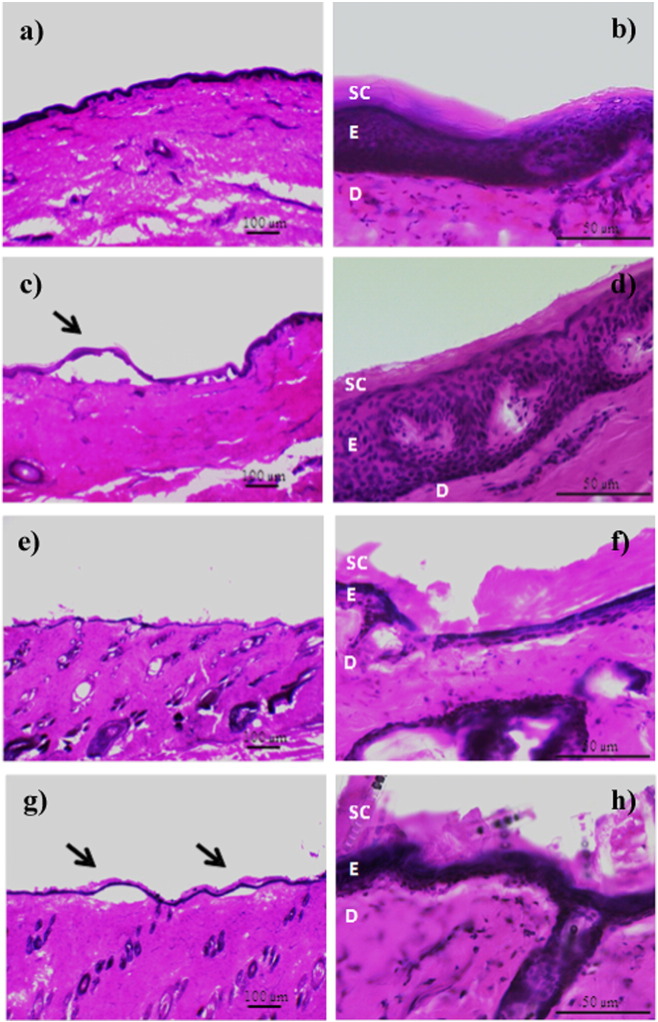
Porcine and rat skin histology a), e) and b), f) control under atmospheric conditions 4 × and 40 × respectively c) and d) porcine skin after hypobaric treatment of 500 mBar for 7 h at 4 × and 40 × respectively g) and h) rat skin after hypobaric treatment of 500 mBar for 1 h at 4 × and 40 × respectively. SC, stratum corneum, E, epidermis, D, dermis and arrow indicates dermal–epidermal detachment.

Light microscopy was used to visualize the histological changes to porcine and rat skin upon hypobaric treatment. Dermal–epidermal detachment was observed in few localized areas of both porcine (Fig. 5c) and rat skin samples (Fig. 5g). However, the majority of the skin maintained its morphological and structural characteristics (Fig. 5d and h) when compared to the control samples (Fig. 5b and f). Minor changes were observed in cell cohesion, with some areas of the *stratum corneum* detaching or peeling off from the adjacent layer. However, this was most probably due to sample handling since this was also observed in the control skin samples.

Such a small amount of dermal–epidermal detachment was thought unlikely to influence drug delivery *via* the skin as the *stratum corneum* seemed to remain intact upon hypobaric driven delivery. Previous *in vivo* work in human subjects has shown a rapid repair of the dermal–epidermal adherence after 2 h of hypobaric treatment [47], [48]. This process was also observed in porcine and rat animal models [49], [50]. The data generated in the current work suggested that the enhanced permeation in response to hypobaric stress was not a consequence of skin damage.

### 3.2 *In vivo* studies

#### 3.2.1 Blood flow

**Fig. 6:**
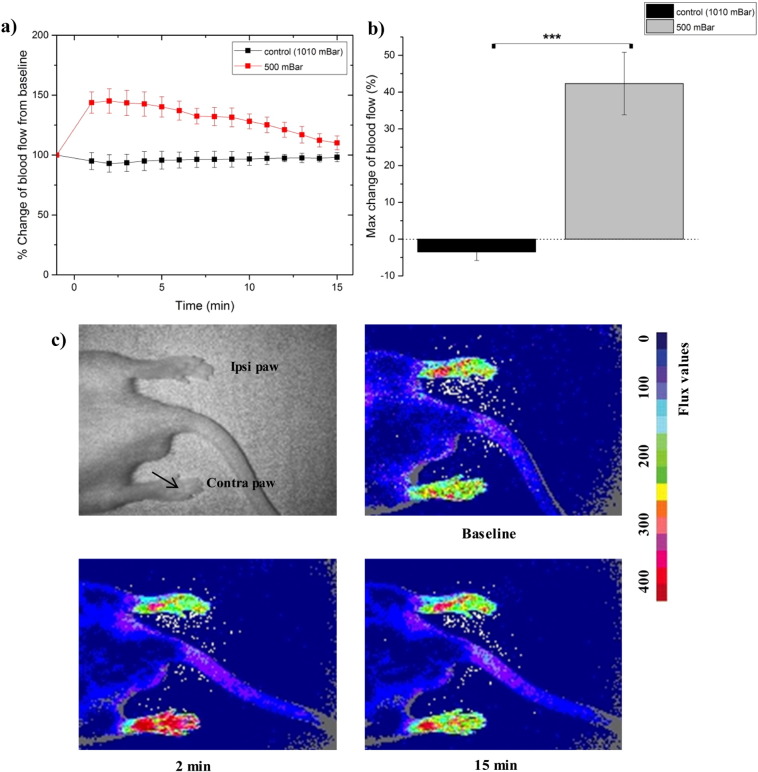
Hypobaric stress induced vascular response a) representative % change in contralateral and ipsilateral (control) hind paw blood flow from baseline over a 15 min period following hypobaric stress treatment b) % change in contralateral and ipsilateral (control) paw blood flow from baseline 3 min after hypobaric treatment (maximum vasodilatation). Student's *t*-test with ***p < 0.001 c) representative full-field laser perfusion imaging pictures alongside grey scale picture showing blood flow at baseline, 2 and 15 min after hypobaric stress treatment of the contralateral hind paw. Arrow indicates the site of topical hypobaric treatment.

The hemodynamic response immediately after the application of hypobaric pressure resulted in a significant increase in blood flow (*p* < 0.001). This effected started to diminish at *ca*. 5 min, which was in good agreement with previously reported maximum hemodynamic response period under hypobaric stress [14] (Fig. 6a). The maximum vasodilation was observed between 0 to 3 min following application of sub-atmospheric pressure to the contralateral hind paw (Fig. 6b and c). This response was significantly greater (*p* > 0.05) with a 42.3 ± 8.5% increase in the contralateral hind paw blood flow upon hypobaric treatment, when compared to the control (Fig. 6c). The increase in cutaneous blood flow upon the application of sub-atmospheric pressures was attributed to a pressure gradient between the surrounding tissues and the site of topical mechanical stress [51], [52].

#### 3.2.2 Pharmacokinetic studies

**Fig. 7:**
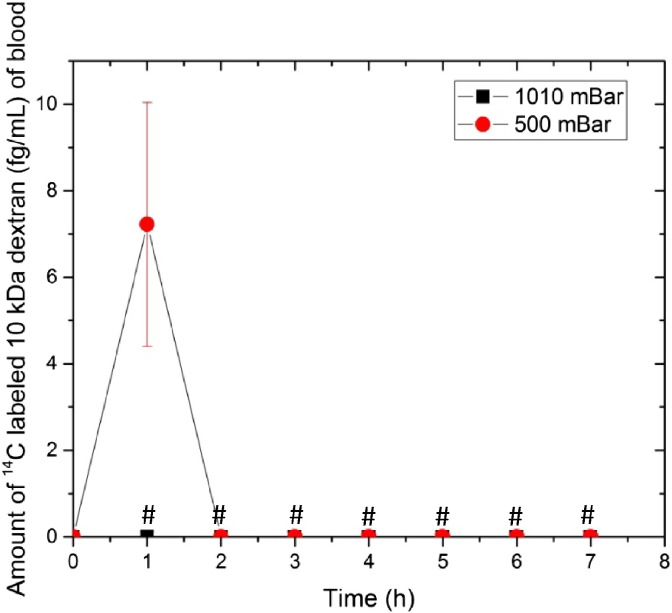
Blood concentration *vs*. time profile of ^14^C-labeled 10 kDa dextran in phosphate buffer (0.79 μCi equivalent to 1.428 pM) applied topically under atmospheric (1010 mBar) and hypobaric conditions (500 mBar). Each point represents mean ± standard deviation (n = 5). ^#^Values below limit of detection (< 3 × background level measurements).

The FD-10S was selected as the model macromolecule for the *in vivo* percutaneous studies since the results from the *in vitro* data showed that the larger molecule was enhanced to a greater extent by hypobaric pressure. *In vivo*, the topical application of local hypobaric stress resulted in a rapid appearance of the dextran in the systemic circulation after 1 h (Fig. 7, 7.2 ± 2.81 fg·mL^− 1^). However, as the systemic levels of dextran for the remainder of the time course were found to be too low to be accurately quantified by liquid scintillation counting, parameters such as C_max_ and T_max_ could not be derived.

The *in vivo* transdermal delivery enhancement of the FD-10S upon the application of topical hypobaric stress appeared to accord with the *in vitro* skin characterization results. It was notable that the permeation was only enhanced during the application of the hypobaric stimuli. This concurred with the hypothesis that the hypobaric pressure was temporally opening the follicular route for this molecule to penetrate into the skin. This follicular opening appeared to quickly reverse when the skin stretching was terminated upon the return to normal pressure conditions and therefore this method of percutaneous penetration enhancement could be considered as being reversible, *i*.*e*., it did not damage the barrier properties of the skin.

The cutaneous deposition profile of dextran supported the pharmacokinetic analysis and the previous *in vitro* studies. Drug deposition was found to be significantly greater (*p* < 0.01, data not displayed graphically) under hypobaric pressure conditions in each layer of the skin, but this effect was more pronounced upon dextran localization in deeper skin tissues. The amount of dextran found in the *stratum corneum* and dermal tissue was 4.3 and 5.7-fold higher, respectively (data not shown graphically).

**Fig. 8:**
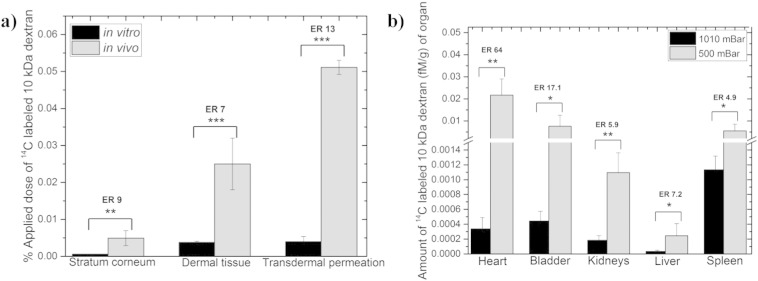
*in vivo* percutaneous penetration studies under atmospheric (1010 mBar) and hypobaric (500 mBar) pressure conditions. a) *in vitro vs*. *in vivo* 10 kDa dextran rat skin deposition b) biodistribution of ^14^C labeled 10 kDa dextran. Each point represents mean ± standard deviation (n = 5). ER (Enhancement ratio) represents the ratio between the amount of drug found under hypobaric and atmospheric conditions. Students *t*-test with *p < 0.05, **p < 0.01 and ***p < 0.001.

I*n vivo* transdermal delivery was found to be significantly higher (*p* < 0.001) when compared to that observed *in vitro* (Fig. 8a) and it resulted in a 13-fold increase in the amount of drug that permeated through the skin when the cutaneous microcirculation was present despite. This was unusual because the skin barrier is commonly thought to be more permeable when tested *in vitro* [18]. The enhanced *in vivo* uptake was thought to be as result of the significant effect of the skin microcirculation on the uptake of the dextran. This result was therefore considered as evidence to suggest that in addition to the hypobaric pressure opening the follicular pathway in the skin the increase in cutaneous blood flow had a significant role on enhancing macromolecule deposition in the cutaneous tissue. This hypothesis accords with previous work, which suggested that an enhanced blood flow allowed deeper tissue distribution of topically applied agents [53], [54], [55]. There could have been some synergy between the follicular transport enhancement and the increase in blood flow because the follicular structures are heavily vascularised, but this could not be confirmed with the current data set [56], [57]. Organ uptake of the ^14^C-labeled 10 kDa dextran following topical delivery (Fig. 8b) supported the blood sampling and cutaneous tissue results in that it showed higher levels upon the application of hypobaric stress. A greater tissue uptake (*p* < 0.05) under hypobaric driven delivery was observed in all the collected organs, which was thought to be attributable to a greater dextran uptake into the systemic circulation induced by the pressure applied to the skin surface. The distribution of the dextran was consistent with its high molecular weight and this provided support for the earlier suggestion that the molecule had not been chemically modified during the delivery process.

## 4 Conclusions

The application of local hypobaric stress appears to be simple yet effective method to increase the percutaneous penetration of topically applied macromolecules. In this study the pressure was applied to the skin using a depressurised chamber that created an airtight seal with the surface of the skin and this same technique could be applied to humans by trained medical professionals. However, some development of the application device would be needed to make it portable and thus suitable for home use if this was required. The application of hypobaric stress causes a series of changes to the skin, but considering both the spectroscopic data and the transport data the two most significant effects of local pressure appeared to be the increase in blood flow and the enlargement of the follicular infundibula. In combination, these two effects seemed to promote the transport of dextran molecules into the skin and their passage into the systemic circulation. The maximum size of dextran used in this study was 10,000 Mw and therefore the maximum size of molecule that hypobaric pressure could drive into the skin is unknown. Further work is needed to understand if hypobaric stress could deliver larger molecules such as vaccines into the skin and to validate this penetration enhancement technique in man. In addition, the skin's response to repeated application of hypobaric stress should be assessed *in vivo* if this novel technology is to be used in the treatment of chronic conditions.
