# Supplementary material for: The application of local hypobaric pressure — A novel means to enhance macromolecule entry into the skin
Source: J Control Release. 2016 Mar 28;226:66–76. doi: 10.1016/j.jconrel.2016.01.052 (PMC4819566; doi:10.1016/j.jconrel.2016.01.052)
Supplement: Supplementary file 1 — Supplementary figures [file mmc1.docx]

**Supplementary data**

**d**


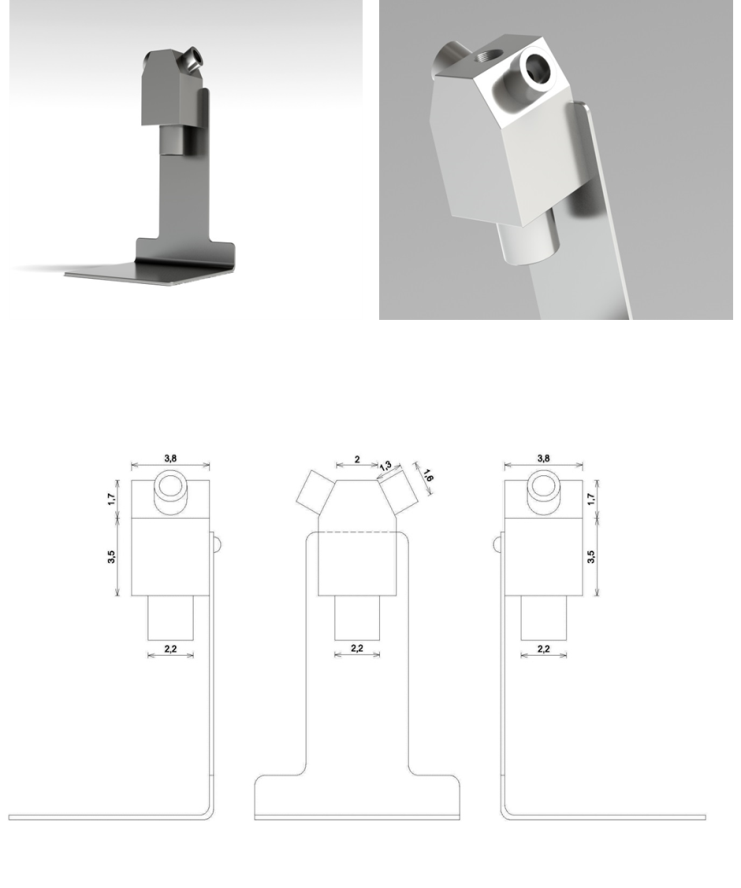


**a**

**b**

**c**


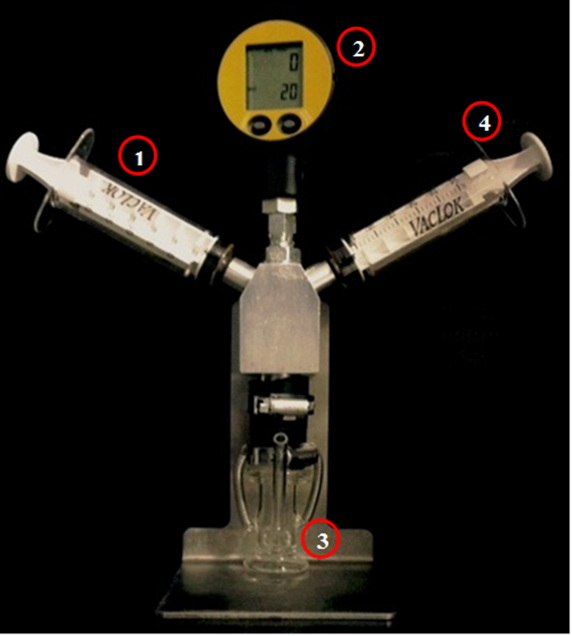


***Figure S1. In-house developed pressure cell****. 3 D and 2 D drawings of the designed aluminium support frame generated in AutoCad LT software (Autodesk, Farnborough, UK), a) front view, b) hypobaric chamber, c) front and lateral views with measurements presented in cm, d) pressure cell set up. Hypobaric pressure was generated by removing a known volume of air using a syringe (1) and changes in hypobaric pressure were recorded with a manometer (2) (Omni Intruments Ltd., Dundee, UK). The system was not completely airtight as the recover chamber sampling port of the standard Franz diffusion cell (3) was left open and hence, hypobaric pressure was shown to decrease overtime (183 ± 5.8 mBar over 420 min). A second syringe (4) was used to correct for pressure decrease during the experimental period at ca. 45 min intervals resulting in a hypobaric pressure that displayed a 15 ± 4.8% variance during the experiments.*


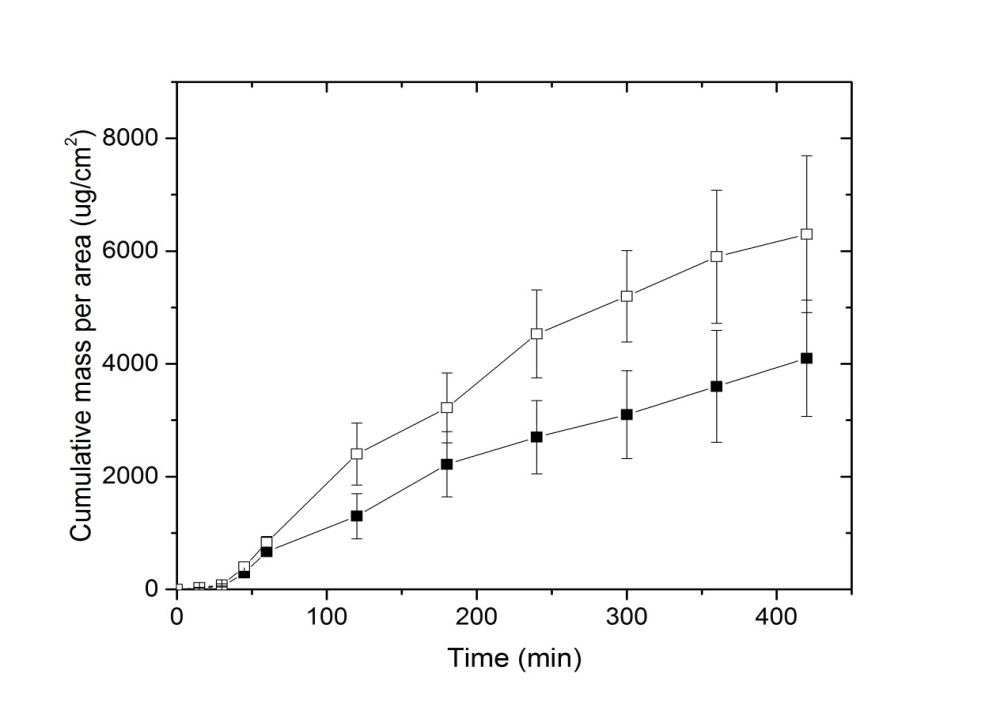

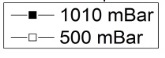

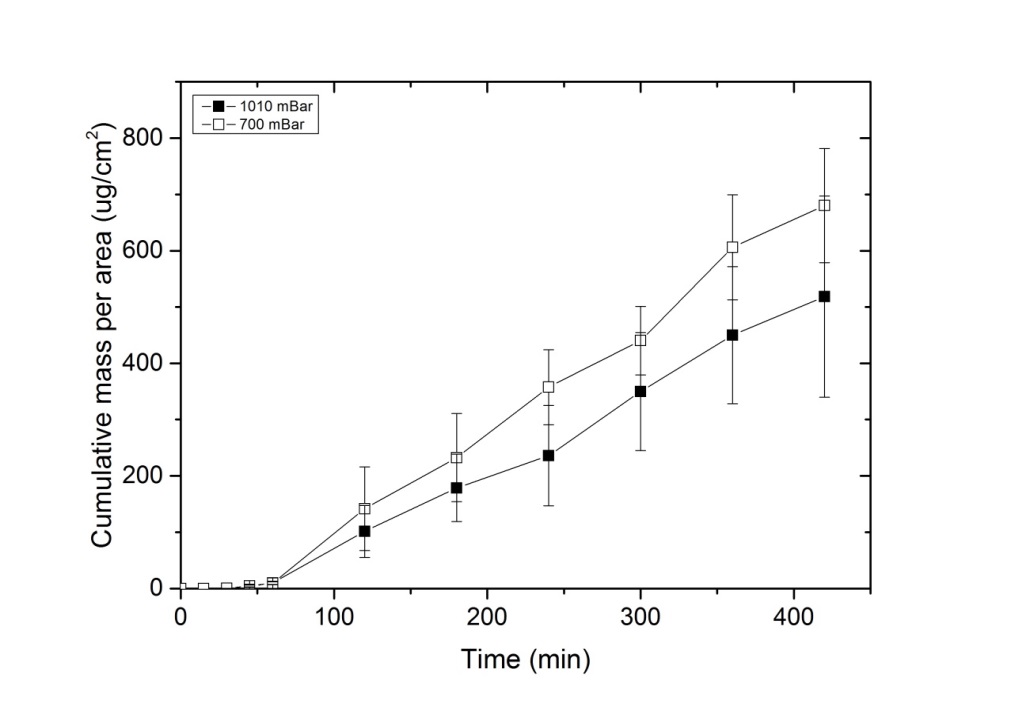

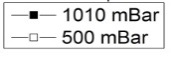


**a)**

**b)**

***Figure S2. Hypobaric pressure permeation studies protocol development.*** *Tetracaine permeation profile under atmospheric (1010 mBar) and hypobaric (500 mBar) pressure through a) silicone membrane and b) porcine skin over 7 h. Each point represents mean ± standard deviation (n = 5).* *The transport data showed that tetracaine steady-state permeation was significantly greater (p < 0.001) when the silicone membrane was challenged under hypobaric stress, but this effect was not mirrored in the porcine skin and the rate of mass transfer was found to be statistically equivalent (p > 0.05). The lack of change in tetracaine permeation through porcine stressed skin was attributed in these preliminary studies to solvent back diffusion into the donor fluid, which was visually apparent at the end of the experimental period. This led to the use of a sponge that functioned as the receiver compartment in the subsequent studies as this negated the back diffusion effects. The observed back flow was thought not to be caused by a disruption of the mechanical integrity of the skin induced by hypobaric treatment, but a consequence of the presence of a large volume of liquid under the skin which did not represent in vivo conditions.*

*
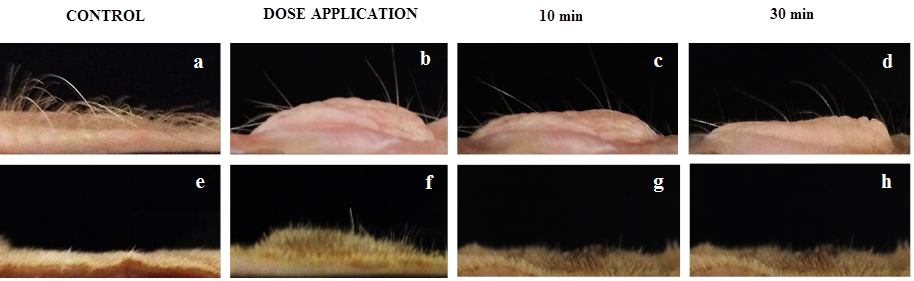
*

***Figure S3***. ***Vertical displacement of porcine (PS) and rat skin (RS) upon the application of the hypobaric conditions employed in the permeation studies*** *a) PS control under atmospheric conditions (1010 mBar) e) RS control under atmospheric conditions (1010 mBar) b) PS immediately upon the application of 500 mBar for 7 h, f) RS immediately upon the application of 500 mBar for 1 h, c) and g) PS and RS after 10 min of hypobaric stress d) and h) PS and RS after 30 min of hypobaric stress.*
